# Cost-utility and budget impact analyses of cervical cancer screening using self-collected samples for HPV DNA testing in Thailand

**DOI:** 10.1186/s12889-023-17358-0

**Published:** 2023-12-04

**Authors:** Chayanis Kositamongkol, Sukrit Kanchanasurakit, Euarat Mepramoon, Pattarawalai Talungchit, Pattama Chaopotong, Kirati Kengkla, Thanet Chaisathaphol, Surasak Saokaew, Pochamana Phisalprapa

**Affiliations:** 1 Division of Ambulatory Medicine, Department of Medicine, Faculty of Medicine Siriraj Hospital, Mahidol University, Bangkok, Thailand; 2 Division of Pharmacy Practice, Department of Pharmaceutical Care, School of Pharmaceutical Sciences, University of Phayao, Phayao, Thailand; 3 Division of Pharmaceutical Care, Department of Pharmacy, Phrae Hospital, Phrae, Thailand; 4 Center of Health Outcomes Research and Therapeutic Safety (Cohorts), School of Pharmaceutical Sciences, University of Phayao, Phayao, Thailand; 5 Unit of Excellence on Clinical Outcomes Research and IntegratioN (UNICORN), School of Pharmaceutical Sciences, University of Phayao, Phayao, Thailand; 6 Department of Obstetrics and Gynecology, Faculty of Medicine Siriraj Hospital, Mahidol University, Bangkok, Thailand

**Keywords:** Budget impact, Cervical cancer, Cost-utility, HPV, Human papillomavirus, Policy, Screening

## Abstract

**Introduction:**

Cervical cancer ranks as the third most prevalent cancer among women in Thailand. However, the effectiveness of cervical cancer screening programs is limited by several factors that impede the screening rate. The utilization of self-collected samples for screening purposes has the potential to alleviate barriers to screening in Thai women. This study assessed the cost-utility and budget impact of implementing cervical cancer screening using self-collected samples for human papillomavirus (HPV) deoxyribonucleic acid (DNA) testing in Thailand.

**Materials and methods:**

We employed a decision tree integrated with a Markov model to estimate the lifetime costs and health benefits associated with the cervical cancer screening program for women aged 25–65. The analysis was conducted from a societal perspective. Four screening policy options were compared: (1) additional self-collected samples for HPV DNA testing, (2) clinician-collected samples for HPV DNA testing only, (3) clinician-collected samples for cytology test (i.e., status quo), and (4) no screening. The model inputs were based on unvaccinated women. The screening strategies and management in those with positive results were assumed followed to the Thai clinical practice guideline. Costs were reported in 2022 Thai baht. Sensitivity analyses were conducted. The ten-year budget impacts of the additional self-collected samples for HPV DNA testing were calculated from a payer perspective.

**Results:**

All screening policies were cost-saving compared to no screening. When comparing the additional self-collected samples for HPV DNA testing with the clinician-collected samples policy, it emerged as the dominant strategy. The incremental benefit in cervical cancer prevention achieved by incorporating self-collected samples for screening was observed at any additional screening rate that could be achieved through their use. Sensitivity analyses yielded consistently favorable results for the screening policies. The average annual budget impact of the additional self-collected samples for screening policy amounted to 681 million Thai baht. This budget allocation could facilitate cervical cancer screening for over 10 million women.

**Conclusions:**

An addition of self-collected samples for HPV DNA testing into the cervical cancer screening program is cost-saving. The benefits of this screening policy outweigh the associated incremental costs. Policymakers should consider this evidence during the policy optimization process.

**Supplementary Information:**

The online version contains supplementary material available at 10.1186/s12889-023-17358-0.

## Introduction

Cervical cancer poses a significant health burden in low- and middle-income countries, including those in Southeastern Asia [1, 2]. In Thailand, it ranks as the third most prevalent cancer. An age-standardized incidence rate, in 2020, was 16.4 per 100 000 women [3]. Cervical cancer accounts for nearly 10% of all new cancer cases in Thailand [3]. Moreover, the age-standardized mortality rate associated with the disease in Thailand (7.4 deaths per 100 000 women) [3] is relatively high compared to that of high-income countries [4]. The global strategy for cervical cancer elimination [2] has three key goals. They are (1) vaccinate 90% of girls against human papillomavirus (HPV), (2) screen 70% of eligible women, and (3) provide treatment to 90% of women with positive screening results [2]. The screening policy remains a crucial strategy for cervical cancer elimination in Thailand. However, Thailand’s national immunization program only began including the HPV vaccine for Thai girls in 2017 [5]. The current HPV vaccine coverage is meager. The experts in the field mentioned that due to the vaccine supply shortage during the COVID pandemic, only approximately 10% of Thai girls received at least one dose of the vaccine.

Thailand’s national cervical cancer screening policy encompasses various screening methods, including the Papanicolaou test (Pap smear), visual inspection with acetic acid (VIA), and HPV DNA testing [6]. These methods are provided free of charge to Thai women as part of the national screening program. Despite the implementation of this policy in 2005, the desired reduction in cervical cancer incidence and mortality has not been adequately achieved [6]. The success of cervical cancer screening policies in low- and middle-income countries is hindered by several factors, including the performance of screening tests and the screening rate. The World Health Organization (WHO) recommends using HPV DNA testing as the primary screening test in the general population due to its superior screening accuracy. In 2020, Thailand transitioned from Pap smears and VIA to HPV DNA testing as the primary screening test for Thai women [6].

The screening rate in the United States exceeds 80% among eligible women [1]. In comparison, low rates have been reported in many low- and middle-income countries [7]. The maximum adequate screening rate (i.e., screening at least once in 3 years) in Thailand has reached only approximately 40% [5]. Previous studies have identified various factors that influence screening rates. For instance, women are more likely to undergo screening if the costs are low or free. Their knowledge levels regarding the disease and the importance of screening are also significant determinants [8]. The nature of clinician-collected samples used in traditional screening methods, which lack privacy and can cause embarrassment, pain, and discomfort, also poses significant barriers to screening for Thai women [8]. In this regard, we surmised that self-collected samples for HPV DNA testing would encourage more women to undergo screening.

The WHO guidelines [2] and numerous studies [9–11] have confirmed that self-collected samples for HPV DNA testing yield screening accuracies comparable to those of clinician-collected samples. In January 2022, Thailand initiated a pilot screening campaign that introduced self-collected samples for HPV DNA testing as an option for Thai women who prefer not to undergo clinician-performed screening. This pilot project covered the screening costs for approximately 80 000 women. However, in the long run, including this additional benefit package would incur higher costs and impact the country’s budget allocation strategy. Therefore, conducting an economic evaluation and budget impact analysis is crucial to confirm the feasibility and sustainability of this policy. Consequently, this study aimed to evaluate the cost-utility and budget impact of cervical cancer screening using self-collected samples for HPV DNA testing in Thailand.

## Materials and methods

### Overall description

A cost-utility analysis was conducted to estimate the lifetime costs and health benefits of incorporating self-collected samples for HPV DNA testing into the cervical cancer screening policy. The analysis was conducted based on unvaccinated women only. Even if HPV DNA testing is currently encouraged to be used as the primary screening method in Thailand, majority of Thai women underwent cervical cancer screening by a cytology test (Pap smear). Consequently, the study framework compared three screening policies: one using “clinician-collected samples for cytology test”, another using “clinician-collected samples for HPV DNA testing”, and the other using both clinician-collected and self-collected samples for HPV DNA testing (i.e., the “additional self-collected samples for HPV DNA testing)”. The screening frequencies were once in 2 years for women with negative results from a cytology test, once in 5 years for women with negative results of HPV DNA testing, and once a year for women with positive results [12]. A no-screening option was also included for validation purposes. The scope of the study was determined in consultation with clinical experts, policymakers, methodological experts, and payer sectors from university hospitals, secondary healthcare centers, and relevant fields.

Most international cervical screening guidelines recommend screening for women aged 25–65 [2, 13]. However, the current Thai clinical practice guideline [12] suggests screening for women aged 30–65 unless they are at risk of HPV infection (e.g., had sexual intercourse before age 30). Therefore, we analyzed two scenarios. The base-case analysis included women aged 25 and older with a screening age of 25–65. The scenario analysis involved women aged 30 and older with a screening age of 30–65. Additionally, we estimated the 5-year and 10-year financial burden through a budget impact analysis from the perspective of the payer.

This article adheres to the reporting guidelines of the Consolidated Health Economic Evaluation Reporting Standards (CHEERS) Statement [14]. The study protocol was authorized by the Siriraj Institutional Review Board, Faculty of Medicine Siriraj Hospital, Mahidol University (protocol code: 1052/2564[IRB1]; approval number: Si 046/2022).

### Economic model

**Fig. 1:**
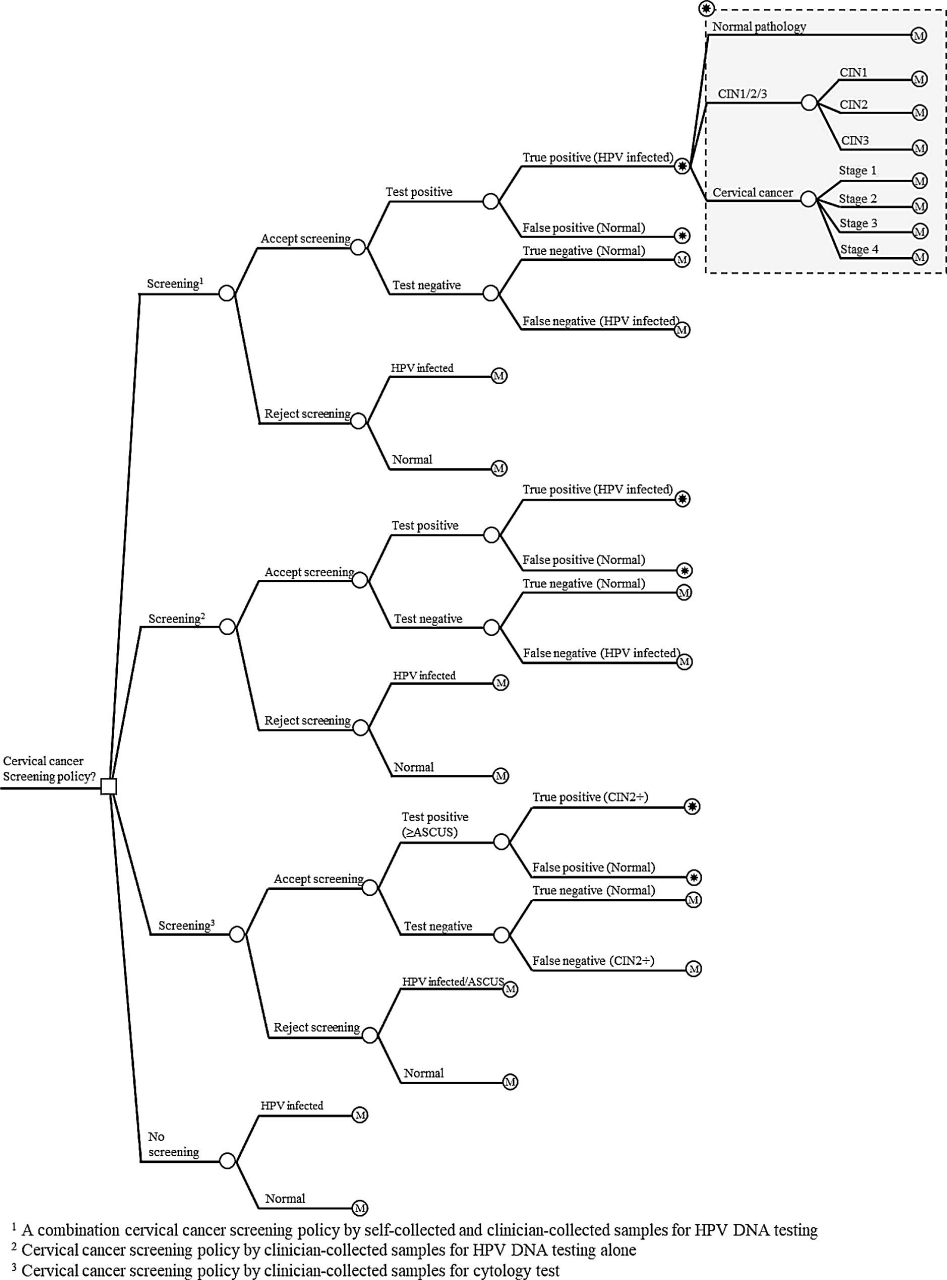
Decision tree

**Fig. 2:**
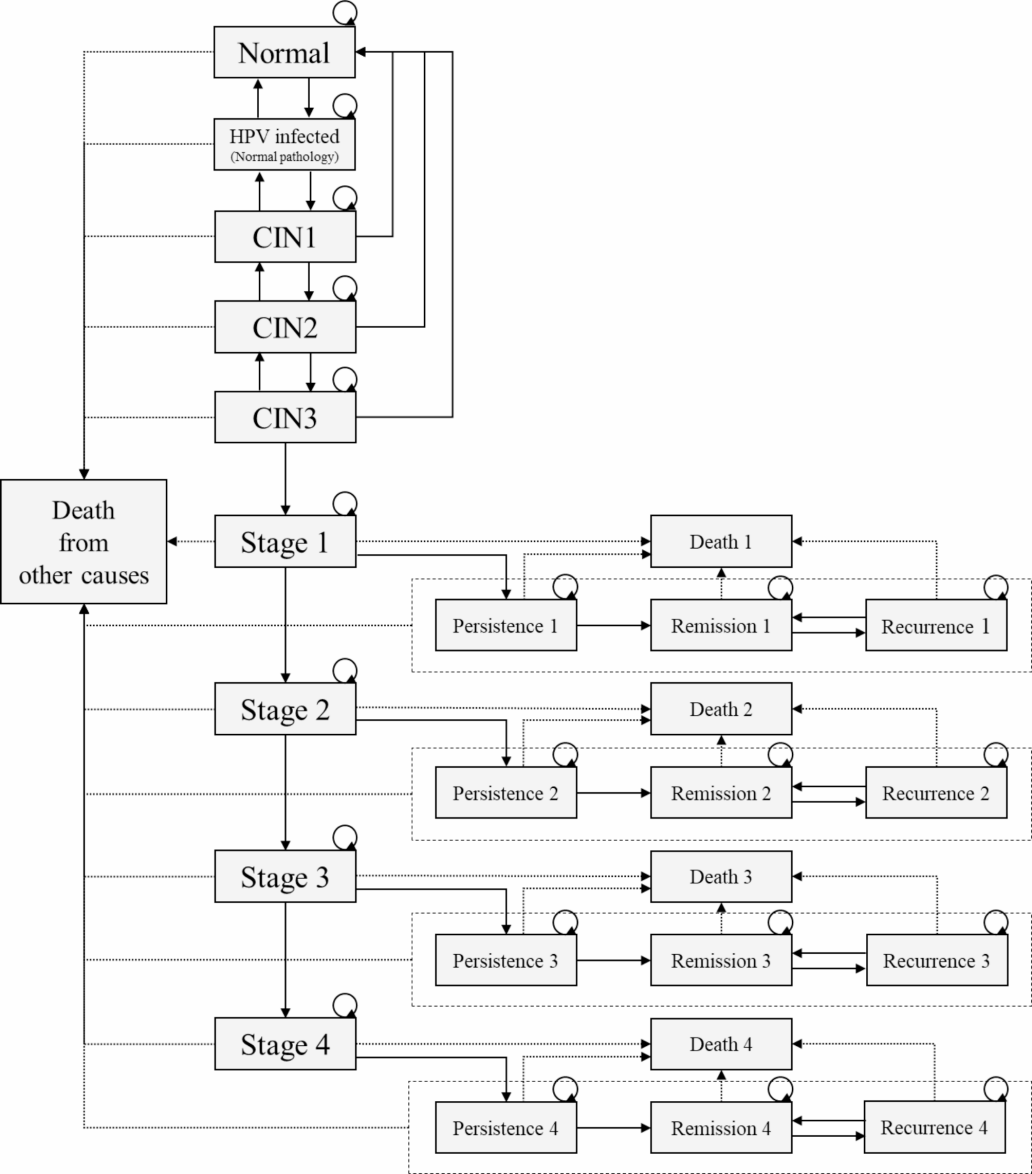
Markov model

A decision tree (Fig. 1) was employed to incorporate screening rates and the performance (sensitivity and specificity) of the screening tests in the model. A Markov model (Fig. 2) was utilized to capture total costs and health outcomes over a patient’s lifetime. The analysis was conducted from a societal perspective and employed a lifetime time horizon, as recommended in Thailand’s Health Technology Assessment guidelines [15]. Both future costs and future outcomes were discounted at an annual rate of 3% throughout the remaining life expectancy of the women [15]. Model input parameters were based on unvaccinated women. The reported outcomes were total lifetime costs (in 2022 Thai baht), total lifetime quality-adjusted life-years (QALYs), the number of prevented incidence cases, the number of averted deaths, and the number needed to screen.

### Interventions

The analysis compared four policy options: (1) additional self-collected samples for HPV DNA testing, (2) clinician-collected samples for HPV DNA testing only, (3) clinician-collected samples for cytology test (i.e., status quo), and (4) no screening. The first option involved HPV DNA testing with clinician-collected samples for women opting for clinician screening and self-collected samples for those preferring not to be screened by a clinician. The analyses were conducted based on scenarios involving self-sampling either at home or in a healthcare setting. The second policy option involved screening with HPV DNA testing using clinician-collected samples exclusively. The third option was added into the model to reflect the status quo of cervical cancer screening policy in Thailand, which majority of women were screened by cytology test.

Our premise was that screening would be undertaken every 5 years for women with negative results from HPV DNA testing, every other year for women with negative cytology test results, and annually for those with HPV infection. In women with positive results from cytology tests, they would be followed up by another cytology test at 6 months. These intervals were followed to the guideline [12]. There would also be no switching between the sample collection methods for normal women. For women who declined screening, they would be eligible to participate in screening every 5 years if they were in the HPV DNA testing arms and every 2 years if they were in the cytology test arm. The disease progression rate of women rejected to screen would follow the natural course of the disease until they undergo screening. Colposcopy would be recommended for all women with positive test results. In cases of HPV infection without abnormal pathology from colposcopy, we assumed that clinician-collected samples for HPV DNA testing would be the only option. Women diagnosed with cervical intraepithelial neoplasia grades 1–3 (CIN1–3) or stage 1–4 cervical cancer would receive standard management following clinical practice guidelines [12].

### Input parameters

#### Screening rate and accuracies of screening methods

We assumed that the screening rates for clinician-collected sample-based methods (i.e., cytology tests and HPV DNA testing) would be similar. Since data on routine cervical cancer screening rates using HPV DNA testing among Thai women were unavailable, we utilized age-specific rates from a study by Termrungruanglert et al. [5]. These were applied as the screening rates for both clinician-collected samples for cytology test and HPV DNA testing in our model. In our base-case analysis, we anticipated that the availability of self-collected samples for HPV DNA testing would result in a cervical cancer screening rate of 80% among eligible women in Thailand. Therefore, the screening rate using self-collected samples was calculated using the following equation:

$$\eqalign{& screening\,rat{e_{self - collected\,samples}}\left( \% \right) \cr & \,\,\,\,\,\,\,\,\,\,\,\,\,\,\,\,\,\,\,\,\,\, = 80 - screening\,rat{e_{clinician - collected\,samples}}\left( \% \right) \cr}$$

However, since the screening rate using clinician-collected samples for women aged 60–65 was lower than expected, the calculated rate using self-collected samples could potentially exceed a realistic range (65%). Consequently, we applied the same value of 46.1% for both the 55–59 and 60–65 age groups. The percentage of women undergoing follow-up colposcopy was set at 68%, per previous research [5], and we assumed that all women undergoing colposcopy would have pathological results. The screening rates used in the model are presented in Table 1.

**Table 1:** Cervical cancer screening rates in Thai women

Age (years)	Screening rates	
	Clinician-collected samples* [5] (%)	Self-collected samples for HPV DNA testing (%)
25–29	30.0	50.0
30–34	28.3	51.7
35–40	38.2	41.8
41–44	40.5	39.5
45–49	42.7	37.3
50–54	40.2	39.8
55–59	33.9	46.1
60–65	15.0	46.1
Follow-up colposcopy [5]	68%	

^*^Clinician-collected samples for cytology or HPV DNA testing

HPV DNA, human papillomavirus deoxyribonucleic acid

The accuracies of screening methods, both cytology test and HPV DNA testing, were derived from a meta-analysis by Arbyn et al. [10] and a real-world study in routine primary HPV screening in the Netherlands [16, 17]. The study pooled the sensitivities and specificities of clinician-collected samples for HPV tests used for primary cervical cancer screening from 14 studies worldwide. Various sampling devices and HPV assays were used in these studies. The impact of the sampling devices on test accuracy could not be conclusively determined. However, there was minimal variation in accuracy across the studies. The meta-analysis also reported that polymerase chain reaction (PCR)-based HPV DNA tests performed similarly for self-collected and clinician-collected samples [10]. Thus, we calculated the accuracy of self-collected samples for HPV DNA testing based on the relative sensitivity and specificity of validated PCR-based HPV assays. The relative sensitivity and specificity of self-collected samples compared with clinician-collected samples for HPV DNA testing were 0.91 (95% confidence interval [CI]: 0.88–0.96) and 1.02 (95% CI: 1.01–1.02), respectively. The pooled sensitivity and specificity of cytology test and HPV DNA testing used in our analysis are presented in Table 2.

**Table 2:** Epidemiological data, test accuracies, treatment effectiveness, transitional probabilities, and utilities

Parameters/Health states	Values	SE	References
**Epidemiological data**			
Prevalence			
HPV infection at age 25–30 years	11.7%	1.9%	Tangjitgamol, 2022 [18]
HPV infected but normal pathology	56.7%	3.4%	Phoolcharoen, 2017 [19]
CIN2+ (included cervical cancer)	1.7%	0.3%	Phoolcharoen, 2017 [19]
Cervical cancer (per 100 000 women)	68.6	8.3	Globocan, 2020 [3]
Incidence of high-risk HPV infection	0.0051	0.0005	Shama, 2012 [20]
**Accuracies of screening tests for detecting CIN2 or worse (%, 95% CI)**			
Self-collected samples for HPV DNA testing: relative sensitivity	0.91	0.88, 0.96	Inturrisi, 2021 [17], Arbyn, 2022 [16]
Self-collected samples for HPV DNA testing: relative specificity	1.02	1.01, 1.02	Inturrisi, 2021 [17], Arbyn, 2022 [16]
Clinician-collected samples for HPV DNA testing: sensitivity	91%	87, 94	Arbyn, 2014 [10]
Clinician-collected samples for HPV DNA testing: specificity	88%	85, 91	Arbyn, 2014 [10]
Clinician-collected samples for cytology test: sensitivity (test cutoff at ASCUS or worse)	83%	75, 89	Arbyn, 2014 [10]
Clinician-collected samples for cytology test: specificity (test cutoff at ASCUS or worse)	91%	87, 94	Arbyn, 2014 [10]
**Treatment effectiveness**			
Treatment access rate	100%	-	Assumption
% eligibility for treatment for CIN1	90%	-	Campos, 2020 [42]
Treatment effectiveness	88%	9%	Campos, 2020 [42]
Proportion of women retaining HPV infection following treatment	15%	-	Campos, 2020 [42]
Regression rate of CIN2/3 to normal or CIN1/2 with treatment	0.46	0.20	Tainio, 2018 [24]
**Transitional probabilities**			
Progression of condition/disease			
HPV infected → CIN1	0.069	0.015	Praditsitithikorn, 2011 [21]
CIN1 → CIN2	0.155	0.024	Bekos, 2018 [23]
CIN2 → CIN3	0.270	0.065	Bekos, 2018 [23]
CIN3 → stage 1	0.026	0.008	Bekos, 2018 [23]
Stage 1 → stage 2 Probability of recurrence	0.355 0.010	0.296 0.001	Praditsitithikorn, 2011 [21] Gomez-Hidalgo, 2022 [22]
Stage 2 → stage 3 Probability of recurrence	0.415 0.010	0.296 0.001	Praditsitithikorn, 2011 [21] Gomez-Hidalgo, 2022 [22]
Stage 3 → stage 4 Probability of recurrence	0.495 0.107	0.131 -	Praditsitithikorn, 2011 [21] Xue, 2018 [25]
Stage 4 Probability of recurrence	0.234	-	Scatchard, 2012 [26]
Regression of condition/disease			
HPV infected → normal age 25–29 years age ≥ 30 years	0.370 0.103	0.033 0.018	Praditsitithikorn, 2011 [21]
CIN1 → normal age 25–34 years age ≥ 35 years	0.140 0.071	0.022 0.019	Praditsitithikorn, 2011 [21], Bekos, 2018 [23]
CIN1 → HPV infected age 25–34 years age ≥ 35 years	0.021 0.011	0.002 0.002	Praditsitithikorn, 2011 [21], Bekos, 2018 [23]
CIN2/3 → normal or CIN1/2	0.069	0.013	Praditsitithikorn, 2011 [21]
CIN2/3 → normal or CIN1/2 (with treatment)	0.411	0.113	Tainio, 2018 [24]
Stage 2 Recurrence 2 → remission 2	0.760	0.015	Xue, 2018 [25]
Stage 3 Persistence 3 → remission 3 Remission 3 → remission 3 Recurrence 3 → remission 3	0.760 0.850 0.760	0.015 0.015 0.015	Xue, 2018 [25] Xue, 2018 [25] Xue, 2018 [25]
Stage 4 Recurrence 4 → remission 4	0.220	0.015	Scatchard, 2012 [26]
**Utilities**			
Normal/HPV infected/CIN1 age 25–34 years age 35–44 years age 45–54 years age 55–64 years age ≥ 65 years	0.91 0.89 0.86 0.80 0.78	0.09 0.09 0.09 0.08 0.08	Termrungruanglert, 2021 [5]
CIN2-3 age 25–34 years age 35–44 years age ≥ 45 years	0.91 0.89 0.87	0.09 0.09 0.09	Termrungruanglert, 2021 [5]
Cervical cancer stage 1 Persistence 1 Remission 1 Recurrence 1	0.74 0.80 0.79 0.8	0.01 0.20 0.01 0.03	Praditsitithikorn, 2011 [21]
Cervical cancer stage 2 Persistence 2 Remission 2 Recurrence 2	0.76 0.80 0.79 0.68	0.01 0.04 0.01 0.02	Praditsitithikorn, 2011 [21]
Cervical cancer stage 3 Persistence 3 Remission 3 Recurrence 3	0.72 0.65 0.81 0.66	0.02 0.05 0.01 0.04	Praditsitithikorn, 2011 [21]
Cervical cancer stage 4 Persistence 4 Remission 4 Recurrence 4	0.63 0.45 0.85 0.81	0.03 0.05 0.05 0.08	Praditsitithikorn, 2011 [21]

ASCUS, atypical squamous cells of undetermined significance; CIN, cervical intraepithelial neoplasia; HPV DNA, human papillomavirus deoxyribonucleic acid; SE, standard error

#### Epidemiological data and treatment effectiveness

The prevalence proportions and incidence rates of HPV infection and cervical cancer in Thai women were derived from previous literature. The reported prevalence of HPV infection in Thai women aged 25–30 was 11.7% [18]. The severity of conditions and diseases in women with HPV infection were calculated based on data from a population-based study conducted in suburban areas of Thailand. That study indicated that over half of the women with positive test results had normal pathology results from follow-up colposcopy, while approximately 1.7% were diagnosed with CIN2 or worse [19]. Incidence rates of HPV infection were based on the work of Sharma et al. [20], who aggregated HPV infection incidences from eight primary studies. The reported range of HPV infection incidence was 0.0001–0.01 per year, and we used the mid-range value in our model. Moreover, the country-specific cervical cancer prevalence was 68.6 per 100 000 women, based on data from the Global Cancer Observatory [3].

Regarding inputs related to treatment effectiveness after abnormal detection, we incorporated two key pieces of data into the model. First, we included information on the proportion of women eligible for treatment. Second, we used data on the proportion of women who retained HPV infection following treatment. The treatment access rate was assumed to be 100% due to the universal health coverage policy in Thailand. These inputs are displayed in Table 2.

#### Natural course of cervical cancer and transitional probabilities

The natural course of cervical cancer and transitional probabilities were obtained from Thai and foreign studies. These included research conducted by Praditsitithikorn et al. [21], Gomez-Hidalgo et al. [22], Bekos et al. [23], Tainio et al. [24], Xue et al. [25], and Scatchard et al. [26]. Details are presented in Table 2.

#### Mortality rate

The mortality rate was divided into two categories: (1) deaths from cervical cancer and (2) deaths from other causes. We obtained the mortality rates of women with cervical cancer from a recent study on the epidemiologic and economic impact of the quadrivalent HPV vaccine in Thailand [5]. Additionally, we used age-specific mortality rates for the general female population in Thailand from the WHO Life Table [27]. The disease-specific mortality rate (${\mu }_{D}$) for cervical cancer was calculated using the following equation:

$${\mu }_{D}= {\mu }_{O}-{\mu }_{ASMR}$$

Here, ${\mu }_{D}$ represents the disease-specific mortality rate, ${\mu }_{O}$ refers to the overall mortality rate of women with cervical cancer obtained from the literature mentioned above [5], and ${\mu }_{ASMR}$ denotes the age-, sex-, and race-specific mortality rates from the WHO Life Table [27]. The detailed mortality rates used in the model are described in Supplementary Appendix 1.

#### Utilities

QALY is a measure used to indicate health effect of virtual patients in a cost-utility analysis model [28]. QALY can be calculated using the equation shown below.

$$QALY = time \,in \,the \,health \,state \left(year\right) * utility$$

*Time in the health state* is the number of years spent in the specific health state of the Markov model. *Utility* refers to the value that represents preferences of individuals have for any particular set of health outcomes. Generally, utility ranges from 0 to 1, which 0 means death and 1 means perfect health [28]. The age-specific utilities of normal women and those with precancerous stages (CIN1–3) were derived from the study by Termrungruanglert et al. [5]. The utilities of women with cervical cancer were obtained from a previous economic evaluation of policy options for cervical cancer prevention and control in Thailand by Praditsitthikorn et al. (Table 2) [21].

#### Costs

As the cost-utility analysis was conducted from a societal perspective, both direct medical and direct nonmedical costs were included. These consisted of all costs occurred in every sector including both patients and healthcare providers. However, indirect costs were not included in the analysis. This decision was based on the assumption that any loss or impairment of work ability or leisure activities due to morbidity would be accounted for in the calculation of QALYs. This approach aligns with the recommendation provided by the Health Technology Assessment Guidelines for Thailand [15]. All cost data were reported in 2022 Thai baht (1 United States dollar = 35.07 Thai baht) using the consumer price index [29].

Costs were obtained from the electronic database of Siriraj Hospital and the database of Standard Cost lists for Health Technology Assessment [30], as shown in Table 3. The management costs for women with CIN1–3 and stage 1–4 cervical cancer were obtained from a cohort of 1423 women. These women were diagnosed with ICD-10 codes C53, D06, and N87 at Siriraj Hospital between January 1, 2015, and December 31, 2021. These costs encompassed treatment procedures, pharmacy expenses, laboratory tests, hospitalization, service fees, and other medical costs. The primary data cost analysis details are presented in Supplementary Appendix 2. The price of a self-sampling kit for HPV DNA testing was obtained from Roche Diagnostics company. The price per kit was 280 Thai baht, which included PCR-based DNA detection. Unit costs for a cytology test (Pap smear), pelvic examination, colposcopy, and pathology tests were sourced from the database of Standard Cost Lists for Health Technology Assessment [30].

**Table 3:** Costs

Costs	Values	Ranges	References
Direct medical costs (THB per visit)			
Self-collected sample kit for HPV DNA testing (included PCR)	280	100, 1000	Roche
Pelvic examination	104	100, 149	HITAP, 2009 [30]
Cytology test (Pap smear)	143	115, 171	HITAP, 2009 [30]
Clinician-sampling for HPV DNA testing	104	100, 149	Assumption
PCR-based HPV DNA detection	280	100, 1000	Roche
Colposcopy	373	356, 1000	HITAP, 2009 [30]
Pathology test	224	180, 267	HITAP, 2009 [30]
Disease management costs (THB per year)			
CIN1	38 258	*	Siriraj Hospital database
CIN2	40 581	*	Siriraj Hospital database
CIN3	46 637	*	Siriraj Hospital database
Stage 1	91 604	*	Siriraj Hospital database
Stage 2	106 310	*	Siriraj Hospital database
Stage 3	132 847	*	Siriraj Hospital database
Stage 4	191 628	*	Siriraj Hospital database
Direct non-medical costs (THB per visit)			
Travel to primary care	59	52, 67	HITAP, 2009 [30]
Travel to hospital	158	133, 183	HITAP, 2009 [30]
Postal service fee	37	20, 100	Thai Postal Service
Food	58	47, 70	HITAP, 2009 [30]

^*^Management costs varied using measures of variability, including standard error, 5th percentile, and 95th percentile of the costs per visit. Details are provided in Supplementary Appendix 2

CIN, cervical intraepithelial neoplasia; HPV DNA, human papillomavirus deoxyribonucleic acid; PCR, polymerase chain reaction; THB, Thai baht

The direct nonmedical costs included expenses for food, transportation to healthcare settings, and postal service fees. We simulated two scenarios for self-sampling locations: home-based collection with delivery via the postal service and sample collection at a nearby healthcare setting. In the latter scenario, the model considered transportation costs to the nearby healthcare setting instead of postal service fees. We assumed that the transportation costs would be equivalent to the cost of traveling to primary care.

### Sensitivity analyses

One-way sensitivity analyses were performed by varying the values of input parameters within the 95% confidence interval and ranges indicated in Tables 2 and 3. We also varied the discount rate within the range of 0–6% per annum. The results of the one-way sensitivity analyses are presented as tornado diagrams. We arranged the most to the least influential parameters respectively from the top to the bottom of the diagrams. Additionally, multivariate probabilistic sensitivity analyses were conducted using Monte Carlo simulations with 1000 iterations in Microsoft Excel (Microsoft Corp, Redmond, WA, USA) [31] to explore the uncertainties of the parameters. Beta distributions were assigned to the transitional probabilities and utilities, while gamma distributions were assigned to all cost data [32]. The results of the probabilistic sensitivity analyses are presented as a cost-effectiveness plane.

### Model calibration

We employed three methods for model calibration. First, face validity was assessed through expert and stakeholder meetings. Second, internal calibration was performed by reviewing and verifying the accuracy of the formulas in Microsoft Excel [31]. Finally, external calibration was conducted by comparing the model outputs with observed data reported by the Global Cancer Observatory; the National Cancer Institute, Ministry of Public Health, Thailand; and the other previously published study.

### Budget impact analyses

The budget impact analyses for the additional self-collected samples for the HPV DNA testing policy were conducted from a payer’s perspective. We calculated the budget impacts for both 5-year and 10-year periods. Only costs of screening and costs of investigations in women with positive results were considered. The input parameters in the budget impact analyses were the same as those applied in the cost-utility analyses. Additionally, we incorporated the proportion of women undergoing total abdominal hysterectomy into the calculation of women eligible for cervical cancer screening. Among women with HPV infection, 20% underwent total abdominal hysterectomy [33], while 0.7% of women in the general population underwent the procedure for reasons other than an HPV infection [5]. Data on the Thai female population were obtained from the National Official Statistics Registration System [34], and the population growth rate was calculated from these data (Supplementary Appendix 3).

## Results

### Model outputs

Cervical cancer incidence, death from cervical cancer, completed screening rate, total screening cost, total cancer prevention cost, and total cancer treatment cost are presented in Supplementary Appendix 4. The policy involving the self-collected samples for HPV DNA testing showed the lowest cervical cancer incidence and mortality rates. In the base-case analysis, the rates were 10.3 incident cases and 9.8 cancer-related deaths per 100 000 women. In the scenario analysis, the rates were 12.8 incident cases and 12.0 cancer-related deaths per 100 000 women. The proportion of women undergo screening was 70% in the base-case analysis and 71% in the scenario analysis. The total screening and cancer prevention costs for the policy involving the self-collected samples for HPV DNA testing were approximately 1.8–1.9 million and 30–37 million Thai baht per 100 000 women, respectively. The total cancer treatment cost for the no-screening policy was the highest.

### Cost-utility analyses

#### Base-case analysis (women age ≥ 25, screening age: 25–65)

The total lifetime costs of the no screening, clinician-collected samples for cytology test, clinician-collected samples for HPV DNA testing, and additional home-based self-collected samples for HPV DNA testing were 47 651, 40 124, 38 850, and 33 052 Thai baht, respectively (33 139 for healthcare-setting-based self-sampling). The total lifetime QALYs were 23.49, 23.54, 23.55, and 23.58, respectively. Compared to the no-screening policy, the clinician-collected samples, both for cytology test and HPV DNA testing, were considered cost-saving because they provided QALY gains while achieving lower lifetime costs. When comparing among HPV DNA testing strategies, the additional self-collected samples for HPV DNA testing screening policy proved to be the dominant strategy (Table 4).

**Table 4:** Results of cost-utility analyses

Policy	Life expectancy (years)	Total lifetime cost (THB)	Total lifetime QALYs	Incremental costs (THB)	QALY gained	Interpretation
**Base-case analysis (women age ≥ 25 years, screening age 25–65 years)**						
No screening	57.0	47 651	23.49			
Clinician-collected samples for cytology test (Pap smear)	57.2	40 124	23.54	-7527	0.05	cost-saving^a^
Clinician-collected samples for HPV DNA testing	57.2	38 850	23.55	-1273	0.001	cost-saving^b^
Self- and clinician-collected samples for HPV DNA testing	57.3	33 052	23.58	-5799	0.03	cost-saving^c^
**Scenario analysis (women age ≥ 30 years, screening age 30–65 years)**						
No screening	52.2	55 993	22.36			
Clinician-collected samples for cytology test (Pap smear)	52.4	47 954	22.41	-8039	0.05	cost-saving^a^
Clinician-collected samples for HPV DNA testing	52.4	46 803	22.42	-1151	0.001	cost-saving^b^
Self- and clinician-collected samples for HPV DNA testing	52.5	41 757	22.45	-6045	0.03	cost-saving^c^

^a^ Compared to no screening

^b^ Compared to clinician-collected samples for cytology test

^c^ Compared to clinician-collected-samples-only for HPV DNA testing

HPV DNA, human papillomavirus deoxyribonucleic acid; QALY, quality-adjusted life-year; THB, Thai baht

#### Scenario analysis (women age ≥ 30, screening age: 30–65)

The total lifetime costs of the no screening, clinician-collected samples for cytology test, clinician-collected samples for HPV DNA testing, and additional home-based self-collected samples for HPV DNA testing were 55 993, 47 954, 46 803, and 40 757 Thai baht, respectively (40 885 for healthcare-setting-based self-sampling). The total lifetime QALYs were 22.36, 22.41, 22.42, and 22.45, respectively. The additional self-collected samples for HPV DNA testing screening policy was again the dominant strategy (Table 4).

Two policies were compared: one allowing screening for women aged 25–65 and the other allowing screening only for women aged 30–65. The analysis revealed that the policy involving screening between 25 and 65 years of age provided greater life expectancy and more QALYs with fewer cancer incident cases and deaths from cervical cancer. The total cancer prevention and treatment costs were also lower for the policy allowing women aged 25–65 to screen, despite higher screening costs being incurred (Table 4 and Supplementary Appendix 4).

### Sensitivity analyses

**Fig. 3:**
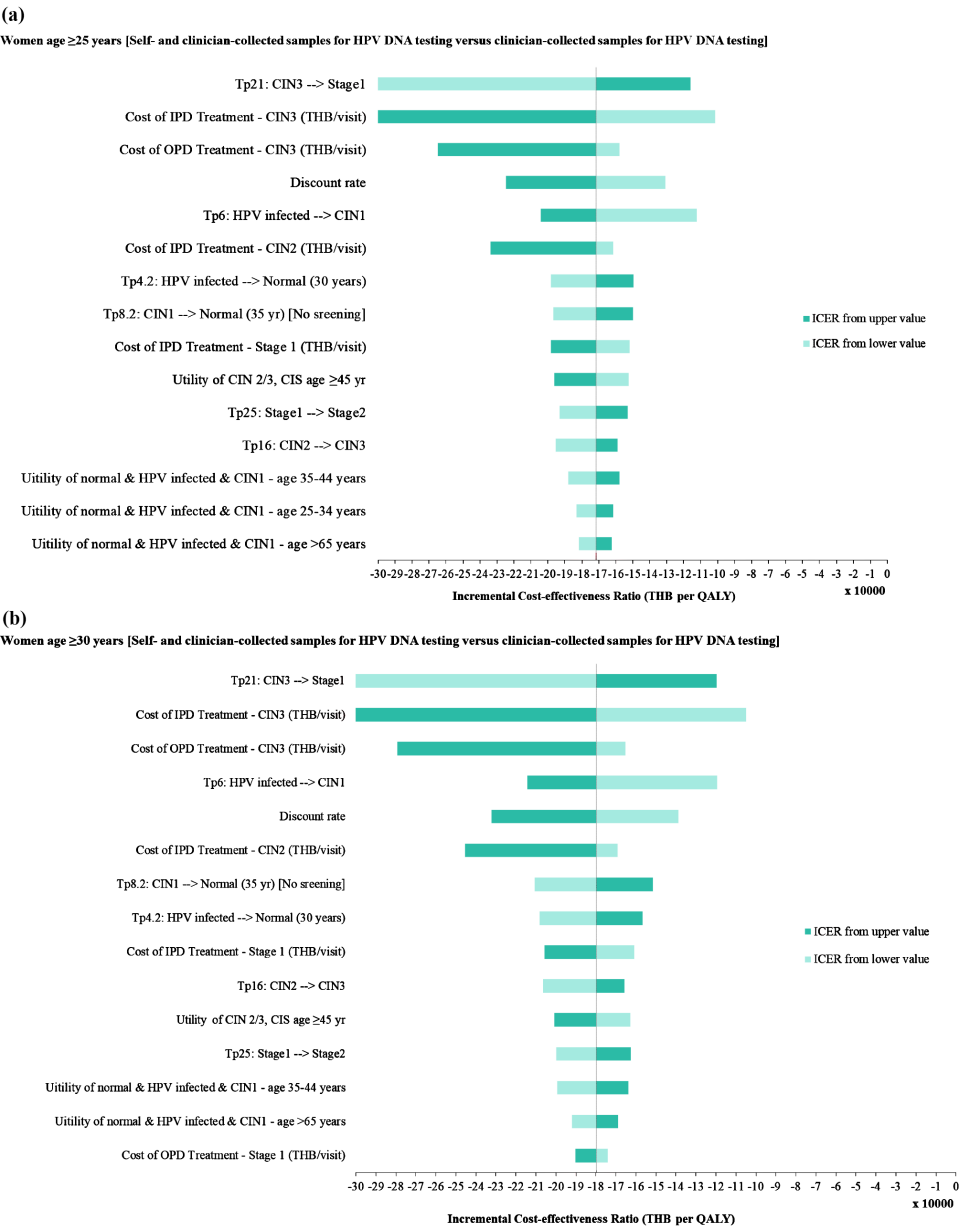
Tornado diagrams of (**a**) base-case analysis and (**b**) scenario analysis. IPD, inpatient department; OPD, outpatient department; THB, Thai baht; Tp, transitional probability; yr, years

One-way sensitivity analyses revealed that the three most influential parameters were the same in the base-case and scenario analyses. They were the transitional probability of CIN3 to stage 1 cervical cancer, the cost of inpatient treatment for CIN3, and the cost of outpatient treatment for CIN3. Moreover, within the range of variation, none of these parameters altered the interpretation of the results (Fig. 3).

**Fig. 4:**
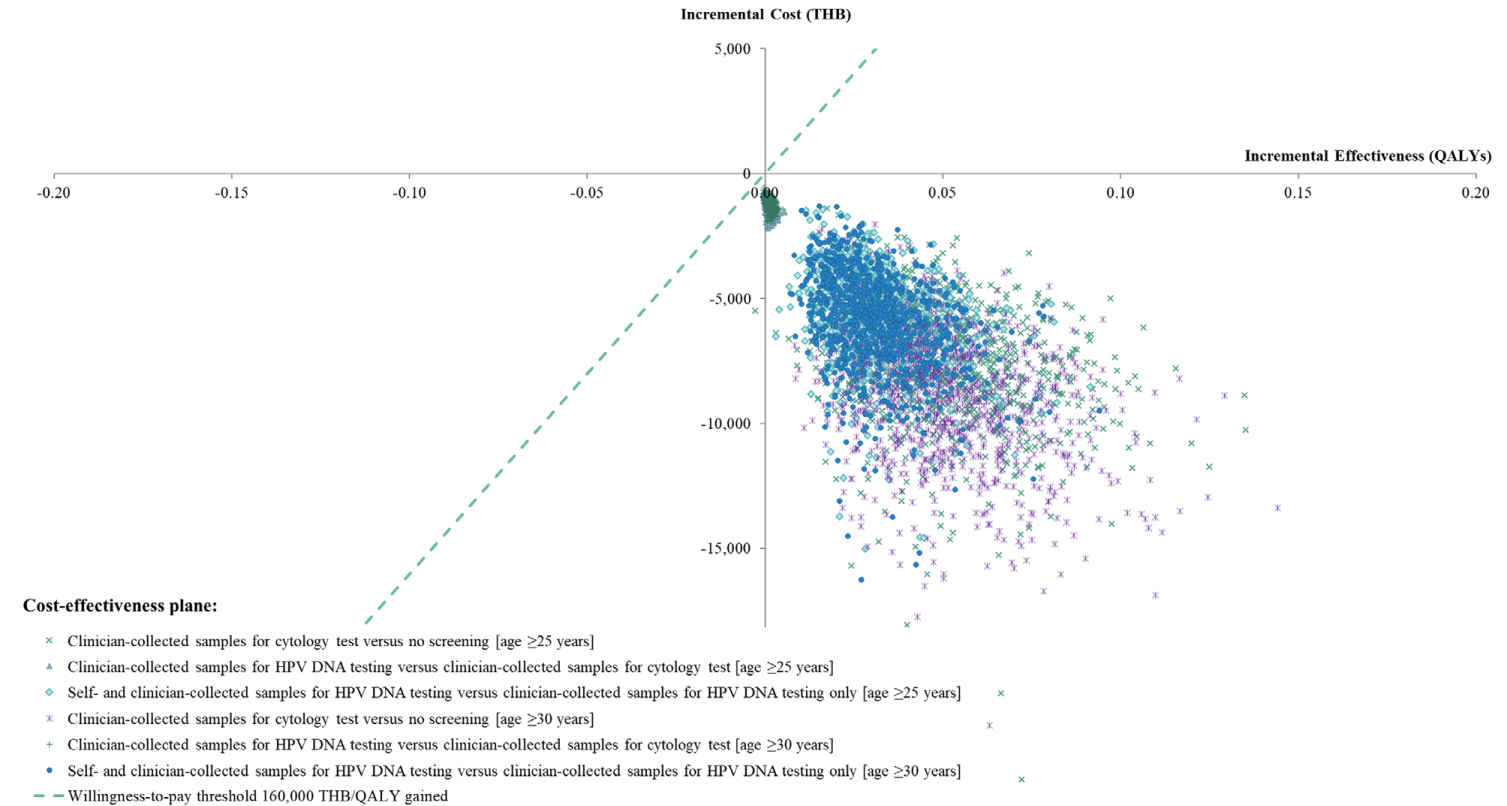
Cost-effectiveness plane

The probabilistic sensitivity analyses demonstrated that the probability of the policy involving additional home-based self-collected samples for HPV DNA testing being cost-saving was 100%. All 1000 iterations in the Monte Carlo simulation demonstrated lower total lifetime costs and higher QALYs than the policies involving clinician-collected samples for HPV DNA testing (Fig. 4).

### Model calibration

The model and its outputs were calibrated through stakeholder meetings involving experts such as obstetricians, gynecologists, healthcare providers, third-party payers, patient sector representatives, and policymakers. The mean cervical cancer incidence of the clinician- collected samples for cytology test from our model was 17.7–20.0 cases per 100 000 women per year. These was comparable to age-standardized incidence rate of 16.4 cases per 100 000 women reported by the Global Cancer Observatory in 2020 [3]. Additionally, the predicted prevalence of HPV infection was comparable to that reported by Praditsitithikorn et al. [21]. The predicted age-specific cervical cancer incidence rates were calibrated by comparing them with data published by the National Cancer Institute, Ministry of Public Health, Thailand [35]. The corresponding plots are shown in Supplementary Appendix 5.

### Budget impact analyses

The average budget impact per year of the policy involving additional home-based self-collected samples for HPV DNA testing was approximately 661–681 million Thai baht. This budget potentially allows approximately 9–10 million women to undergo cervical cancer screening. The details are shown in Table 5.

**Table 5:** Budget impact analyses of cervical cancer screening using HPV DNA testing in women aged 25–65

BIA	# of women eligible for screening	# of women screened	% screened	# of T- women	# of T + women	# of total screening	% of TAH	Total BIA (million THB)	Average per year (million THB)	
**Clinician- and self-collected samples for HPV DNA testing [screening age 25–65 years]**										
5 years	21 657 104	16 785 188	77.5%	14 722 180	2 063 008	18 524 540	2.6%	฿ 6688	฿ 1338	
10 years	23 622 356	18 308 343	77.5%	16 058 129	2 250 213	38 135 036	5.1%	฿ 13 765	฿ 1377	
**Clinician-collected samples for HPV DNA testing**										
5 years	7 287 295	7 287 295	33.6%	6 314 539	972 756	8 113 118	2.9%	฿ 3384	฿ 677	
10 years	7 948 573	7 948 573	33.6%	6 887 546	1 061 028	16 675 645	5.6%	฿ 6956	฿ 696	
**Self-collected samples for HPV DNA testing**										
5 years	14 369 809	9 497 893	43.9%	8 407 641	1 090 252	10 411 422	2.4%	฿ 3304	฿ 661	
10 years	15 673 782	10 359 769	43.9%	9 170 583	1 189 186	21 459 391	4.8%	฿ 6810	฿ 681	

#, number; BIA, budget impact analyses; HPV DNA, human papillomavirus deoxyribonucleic acid; TAH, total abdominal hysterectomy; THB, Thai baht; T+, test positive; T-, test negative

## Discussion

This study in Thailand focused on evaluating the cost-effectiveness and budget impact of a cervical cancer screening policy that utilizes self-collected samples for HPV DNA testing. Past research consistently demonstrates that HPV DNA testing offers a superior screening quality compared to the Pap smear and VIA methods [2, 36]. However, traditional clinician-based screening methods pose obstacles such as embarrassment, inconvenience, pain, and discomfort [37].

Our analyses revealed that implementing additional home-based self-collected samples for HPV DNA testing yielded the lowest total lifetime cost and the highest QALYs among the three policy options examined. Therefore, from a societal perspective, the policy involving additional home-based self-collected samples for HPV DNA testing proved to be cost-saving and the most favorable option.

Furthermore, our study demonstrated that screening women aged 25 and above resulted in more benefits than restricting screening to women aged 30 and above. Screening women aged 25–65 could prevent an additional 2.5 cervical cancer cases per 100 000 women while incurring an extra cost of only around 90 000 Thai baht annually. Moreover, this policy would save approximately 7.4 million Thai baht annually in cancer prevention costs (screening and CIN1–3 treatment costs) compared to limiting screening to women aged 30–65.

Our sensitivity analyses consistently indicated that the policy of additional home-based self-collected samples for HPV DNA testing remained the best option, even when considering the lowest expected screening rate achievable through self-sampling. These results emphasize the effectiveness and cost-saving potential of implementing the self-collected samples for HPV DNA testing for cervical cancer screening in Thailand.

Our findings align with previous studies that have examined the cost-effectiveness of self-collected samples for HPV DNA testing. For instance, a study in Switzerland found that self-collected samples for HPV DNA testing among nonattendees were cost-effective and reduced cervical cancer cases and related deaths [38]. Additionally, a systematic review investigated cervical cancer screening in low- and middle-income countries, examining seven studies on self-collected samples for HPV DNA testing [39]. The review findings revealed the cost-effectiveness of self-collected sample for HPV testing when a higher population coverage was achieved than with other screening methods.

While we did not have direct evidence of screening rates using self-collected sample kits for nonattendees, substantial evidence supports the idea that self-sampling increases population screening coverage [38–40]. The utilization of self-collected samples for HPV DNA testing can help overcome barriers such as embarrassment, inconvenience, price, and test reliability. A study in Thailand showed that the self-sampling was widely accepted even among Muslim women, who constitute approximately 3% of the Thai female population [8]. Moreover, multiple studies and guidelines have confirmed the reliability of HPV DNA testing using self-collected specimens.

Given that HPV DNA testing is covered by all health benefit coverage schemes in Thailand, cost should not hinder screening for Thai women. While some women prefer to collect samples at home, several studies have indicated that others opt to collect their samples in a healthcare setting. This preference stems from valuing the presence of healthcare workers who can offer information and address any concerns they may have [8, 41]. Our study suggests that, from a societal perspective, the outcomes of home-based and healthcare setting-based specimen collection are comparable. Therefore, a revised policy should not be limited to a single strategy but should offer both options to maximize screening rates. However, it is essential to note that self-collected samples for HPV DNA testing cannot wholly replace clinician-based screening methods. The latter methods provide additional benefits by checking for other gynecological problems and catering to women willing to undergo clinician examinations.

Regarding the budget impact, our projections indicate that implementing a policy of self-collecting samples would result in an additional cost of approximately 661–681 million Thai baht per year. While this amount is slightly higher than the current budget for clinician-collected sampling, it is crucial to consider the potential benefits. If the policy permitting the self-collected samples for HPV DNA testing is implemented nationwide, an estimated additional 10 million women will undergo cervical cancer screening, over 10 years. Moreover, the policy will result in the prevention of at least 2000 cases of cervical cancer and 1500 cancer-related deaths per year in addition to what would be achieved by implementing clinician screening alone.

Furthermore, our study demonstrates that initiating screening at the age of 25 would prevent more cervical cancer cases than starting from 30. If there are budgetary, healthcare-workforce, or practicality constraints, the WHO suggests prioritizing cervical cancer screening for women who have never been screened, underscreened women, and women living with HIV [2]. It is important to highlight that the incidence of cervical cancer differs between women aged 25–29 and those above 30. The rates are 6.9 cases per 100 000 women for women aged 25–29 and 12.6 cases per 100 000 women for those above 30 [35]. Additionally, younger women have a higher probability of regression from HPV infection and CIN1 to the normal stage [2, 21]. Policymakers should consider this information when making decisions about cervical cancer screening policies.

To our knowledge, this study represents the first investigation in Thailand to evaluate the cost-effectiveness and budget impact of self-collected samples for HPV DNA testing. A previous study in Thailand focused on comparing the cost-effectiveness of HPV DNA testing using clinician-collected samples to Pap smears [36]. It was concluded that HPV DNA testing was Thailand’s optimal primary cervical cancer screening strategy. Despite the availability of coverage for all screening methods under health benefit schemes in Thailand, the current screening rate among Thai women still needs to be improved.

Our study supports the inclusion of self-collected samples for HPV DNA testing within health benefit coverage schemes, as this option offers greater benefits in cervical cancer prevention. Moreover, allowing self-collected samples for HPV DNA testing proves to be cost-saving compared to relying exclusively on clinician-collected samples.

Several factors contribute to the reliability and contextual relevance of our findings. First, the study framework involved obstetricians, gynecologists, and policymakers from the outset. Second, input parameters were primarily derived from systematic reviews and meta-analyses. Third, sensitivity analyses were conducted by varying parameters in the model, consistently confirming that using additional self-collected samples for testing was the optimal choice. Furthermore, the incremental cervical cancer prevention benefits associated with self-collected samples were observed at any additional screening rate achievable through self-sampling. Last, local data were incorporated into the analyses, ensuring that the results directly apply to policy decisions. Local data on screening rates, cancer incidence, and related costs were utilized whenever possible to ensure that the study accurately reflected the Thai context.

Several limitations to our study should be acknowledged. First, using an economic model with various assumptions have some drawback features. For an example, various individualized characteristic of target women in the virtual cohort could not be captured in the model. Moreover, due to limited data availability, we had to make assumptions about the additional screening rate that would be achieved using self-collected samples. However, sensitivity analyses demonstrated that incremental cervical cancer prevention benefits associated with self-collected samples were observed regardless of the specific additional screening rate.

Second, all costs related to adverse events were included in the treatment costs. Unfortunately, we could not separate these costs from the total treatment costs due to limitations in the database structure. The treatment costs were also based on data from only one university hospital. However, sensitivity analyses indicated that varying the treatment costs within plausible ranges did not change the interpretation that using self-collected samples for testing was the optimal policy option.

Third, our analyses focused on unvaccinated women only since the vaccine coverage rate among Thai girls and women is meager. As more information becomes available on the impact of HPV vaccines, it will be essential to update the model accordingly.

Fourth, our model did not consider the additional benefits of pelvic examinations, such as the detection of other genital disorders. Therefore, we recommend that self-collected samples for HPV DNA testing should be an option only for women who are unwilling to undergo screening by a clinician.

Fifth, various HPV tests with different sampling tools and analysis methods are available on the market, and their performance can vary. Meta-analyses have shown that the accuracy of self-collected samples for HPV testing is lower than that of clinician-collected samples, except when PCR-based DNA detection is used as the analysis method [10]. Thereby, we suggested that PCR-based assay should be an only technique used for analyzing self-collected samples. A systematic review has also suggested that self-collected samples are more cost-effective than clinician-collected samples only when screening coverage increases [39].

Additionally, there are challenges associated with self-collected samples for HPV DNA testing in our specific context. Our models did not consider the costs of public relations, provider training, and patient education. The average level of education in the Thai population is likely to be lower than in countries with a better economic status. Consequently, Thai women may require more guidance to understand the importance of cervical cancer screening. The quality of self-collected samples could also be affected by relatively lower levels of education, potentially leading to sampling errors, delivery errors, and contamination. As a result, our model may have underestimated certain costs associated with policy implementation. However, these fundamental costs related to policy implementation are expected to be short-term.

## Conclusions

All cervical cancer screening policies are cost-saving compared to no screening. The policy involving self-collected samples for HPV DNA testing is the most advantageous option, as it will effectively increase the screening rate. The additional benefits resulting from having dual-collection policies (self-collected samples and clinician-collected samples for HPV DNA testing) in a cervical cancer screening program outweigh the incremental costs of the dual program when compared to a clinician-collected samples for HPV DNA testing only approach. While screening younger women will incur higher upfront screening budgets, it will reduce overall cancer prevention and treatment costs in the long term. Policymakers should consider this evidence during the process of optimizing policies in Thailand.

### Electronic supplementary material

Below is the link to the electronic supplementary material.

Supplementary information regarding model’s inputs and outcomes of the study

## Data Availability

The datasets used and/or analysed during the current study are available from the corresponding author on reasonable request.
