# Supplementary material for: Cost-utility and budget impact analyses of cervical cancer screening using self-collected samples for HPV DNA testing in Thailand
Source: BMC Public Health. 2023 Dec 4;23:2413. doi: 10.1186/s12889-023-17358-0 (PMC10696797; doi:10.1186/s12889-023-17358-0)
Supplement: Supplementary file 1 — Supplementary information regarding model’s inputs and outcomes of the study [file 12889_2023_17358_MOESM1_ESM.docx]

**Cost-utility and budget impact analyses of cervical cancer screening using self-collected samples for HPV DNA testing in Thailand**

**Chayanis Kositamongkol^1^, Sukrit Kanchanasurakit^2–5^, Euarat Mepramoon^1^, Pattarawalai Talungchit^6^, Pattama Chaopotong^6^, Kirati Kengkla^2^, Thanet Chaisathaphol^1^, Surasak Saokaew^2,4–5*^, Pochamana Phisalprapa^1*^**

^1^Division of Ambulatory Medicine, Department of Medicine, Faculty of Medicine Siriraj Hospital, Mahidol University, Bangkok, Thailand

^2^Division of Pharmacy Practice, Department of Pharmaceutical Care, School of Pharmaceutical Sciences, University of Phayao, Phayao, Thailand

^3^Division of Pharmaceutical Care, Department of Pharmacy, Phrae Hospital, Phrae, Thailand

^4^Center of Health Outcomes Research and Therapeutic Safety (Cohorts), School of Pharmaceutical Sciences, University of Phayao, Phayao, Thailand

^5^Unit of Excellence on Clinical Outcomes Research and IntegratioN (UNICORN), School of Pharmaceutical Sciences, University of Phayao, Phayao, Thailand

^6^Department of Obstetrics and Gynecology, Faculty of Medicine Siriraj Hospital, Mahidol University, Bangkok, Thailand

*** Correspondence:**
Pochamana Phisalprapa (coco_a105@hotmail.com);
Surasak Saokaew (surasak.sa@up.ac.th)

Keywords: budget impact, cervical cancer, cost-utility, HPV, human papillomavirus, policy, screening

**Supplementary Appendix 1:** Mortality rates

**Supplementary Appendix 2:** Costs of management of cervical intraepithelial neoplasia and cervical cancer

**Supplementary Appendix 3:** Thai female population

**Supplementary Appendix 4:** Model outputs

**Supplementary Appendix 5:** Model validation

**Supplementary Appendix 1:** Mortality rates

**Table S1** Mortality rates

| **Age (years)** | **ASMR (1)** | **All-cause mortality in women with cervical cancer (2)** | | | **Cervical cancer-specific mortality rate** | | |
| --- | --- | --- | --- | --- | --- | --- | --- |
|  |  | **Stage 1** | **Stage 2-3** | **Stage 4** | **Stage 1** | **Stage 2-3** | **Stage 4** |
| 25-29 | 0.0007 | 0.0990 | 0.1920 | 0.4120 | 0.0983 | 0.1913 | 0.4113 |
| 30-34 | 0.0009 | 0.0410 | 0.3600 | 0.7880 | 0.0401 | 0.3591 | 0.7871 |
| 35-39 | 0.0010 | 0.0410 | 0.3600 | 0.7880 | 0.0400 | 0.3590 | 0.7870 |
| 40-44 | 0.0020 | 0.1070 | 0.3880 | 0.5475 | 0.1050 | 0.3860 | 0.5455 |
| 45-49 | 0.0020 | 0.1070 | 0.3880 | 0.5475 | 0.1050 | 0.3860 | 0.5455 |
| 50-54 | 0.0030 | 0.1575 | 0.3850 | 0.6075 | 0.1545 | 0.3820 | 0.6045 |
| 55-59 | 0.0040 | 0.1575 | 0.3850 | 0.6075 | 0.1535 | 0.3810 | 0.6035 |
| 60-64 | 0.0070 | 0.3300 | 0.4235 | 0.7325 | 0.3230 | 0.4165 | 0.7255 |
| 65-69 | 0.0120 | 0.3300 | 0.4235 | 0.7325 | 0.3180 | 0.4115 | 0.7205 |
| 70-74 | 0.0180 | 0.1820 | 0.4150 | 0.7030 | 0.1640 | 0.3970 | 0.6850 |
| 75-79 | 0.0300 | 0.1820 | 0.4150 | 0.7030 | 0.1520 | 0.3850 | 0.6730 |
| 80-84 | 0.0510 | 0.1820 | 0.4150 | 0.7030 | 0.1310 | 0.3640 | 0.6520 |
| ≥85 | 0.1200 | 0.1820 | 0.4150 | 0.7030 | 0.0620 | 0.2950 | 0.5830 |

ASMR, age-, sex- and race-specific mortality rate

**Supplementary Appendix 2:** Costs of management of cervical intraepithelial neoplasia and cervical cancer

Direct medical costs

Data were extracted from the Siriraj electronic database using the following inclusion criteria:

- Women aged ≥15 years
- ICD-10: C53 (malignant neoplasm of cervix uteri), D06 (carcinoma in situ of cervix uteri), and N87 (dysplasia of cervix uteri)
- Diagnosis period between January 1, 2015, and December 31, 2021

Exclusion criteria included cases with undocumented disease severity and missing data that prevented cost calculations. The Figure S1 shows the number of women used to calculate the costs.

**Figure S1** Number of women used to calculate the costs of cervical intraepithelial neoplasia and cervical cancer management

A total of 1423 women were included in the cost analysis, with an average age of 54.7 ± 14.7 years. The median follow-up time was 1.5 years (interquartile range: 0.4, 3.5). Outpatient department visits totaled 43 295, while inpatient department visits amounted to 4871. The annual number of visits and median costs per visit for each disease stage are presented in Table S2.

**Table S2** Costs of cervical intraepithelial neoplasia and cervical cancer management

| **Health states** | **Number of visits  per year** | **Median costs  (THB per visit)** | **SE** | **Range (P5, P95)** |
| --- | --- | --- | --- | --- |
| Outpatient department | | | | |
| CIN1 | 2 | 268 | 148 | 34, 885 |
| CIN2 | 4 | 818 | 192 | 185, 2978 |
| CIN3 | 5 | 911 | 220 | 46, 6790 |
| Stage 1 | 10 | 2145 | 93 | 381, 5401 |
| Stage 2 | 16 | 3632 | 123 | 502, 6741 |
| Stage 3 | 19 | 3933 | 159 | 684, 7882 |
| Stage 4 | 24 | 3282 | 218 | 768, 8280 |
| Inpatient department | | | | |
| CIN1 | 1 | 33 724 | 9100 | 7209, 69 124 |
| CIN2 | 1 | 26 200 | 24 924 | 13 440, 104 676 |
| CIN3 | 1 | 39 333 | 4663 | 17 863, 88 450 |
| Stage 1 | 1 | 66 401 | 2788 | 8863, 144 817 |
| Stage 2 | 2 | 25 105 | 4095 | 5514, 156 374 |
| Stage 3 | 2 | 26 120 | 4128 | 5001, 161 374 |
| Stage 4 | 4 | 27 921 | 5523 | 5899, 172 960 |

P5, 5^th^ percentile; P95, 95^th^ percentile; SE, standard error

**Supplementary Appendix 3:** Thai female population

**Table S3** Number of Thai female population (3)

| **Age (years)** | **Female population (people)** | **Age (years)** | **Female population (people)** |
| --- | --- | --- | --- |
| 25 | 478 437 | 46 | 531 361 |
| 26 | 488 485 | 47 | 521 791 |
| 27 | 484 247 | 48 | 532 804 |
| 28 | 467 172 | 49 | 523 406 |
| 29 | 472 009 | 50 | 541 371 |
| 30 | 479 796 | 51 | 551 285 |
| 31 | 475 921 | 52 | 538 636 |
| 32 | 471 308 | 53 | 532 115 |
| 33 | 451 904 | 54 | 551 273 |
| 34 | 442 779 | 55 | 524 238 |
| 35 | 443 535 | 56 | 515 287 |
| 36 | 458 754 | 57 | 520 273 |
| 37 | 476 576 | 58 | 519 014 |
| 38 | 480 383 | 59 | 483 306 |
| 39 | 493 445 | 60 | 469 053 |
| 40 | 511 709 | 61 | 447 543 |
| 41 | 511 974 | 62 | 448 965 |
| 42 | 522 686 | 63 | 426 963 |
| 43 | 525 009 | 64 | 393 606 |
| 44 | 510 156 | 65 | 377 736 |
| 45 | 535 634 |  |  |

**Supplementary Appendix 4:** Model outputs

**Table S4** Model outputs

| **Outcomes** | **No screening** | **Clinician-collected samples for cytology test** | **Clinician-collected samples for  HPV DNA testing** | **Self- and clinician- collected samples  for HPV DNA testing** |
| --- | --- | --- | --- | --- |
| **Base-case analysis (women age ≥25 years, screening age 25–65 years)** | | | | |
| Cervical cancer incidence (cases per 100 000 women) | 25.3 | 17.7 | 17.5 | 10.3 |
| Death from cervical cancer (deaths per 100 000 women) | 22.9 | 16.1 | 15.8 | 9.8 |
| Death from other causes (deaths per 100 000 women) | 963.6 | 970.4 | 970.6 | 976.6 |
| Screening rate (%) |  | 26% | 25% | 70% |
| Completed screening rate with follow-up colposcopy (%) |  | 25% | 24% | 68% |
| Total screening cost^a^ (THB per 100 000 women) |  | 1 311 357 | 781 063 | 1 884 813 |
| Total cancer prevention cost^b^ (THB per 100 000 women) | 41 134 051 | 35 620 955 | 34 395 576 | 29 950 372 |
| Total cancer treatment cost^c^ (THB per 100 000 women) | 6 516 748 | 4 502 636 | 4 454 744 | 3 101 396 |
| Cervical cancer incidence^d^ (cases) | 6228 | 4370 | 4308 | 2547 |
| Death from cervical cancer^d^ (deaths) | 5638 | 3962 | 3906 | 2423 |
| **Scenario analysis (women age ≥30 years, screening age 30–65 years)** | | | | |
| Cervical cancer incidence (cases per 100 000 women) | 27.9 | 20.0 | 19.8 | 12.8 |
| Death from cervical cancer (deaths per 100 000 women) | 25.2 | 18.1 | 17.9 | 12.0 |
| Death from other causes (deaths per 100 000 women) | 926.9 | 970.0 | 970.2 | 976.0 |
| Screening rate (%) |  | 25% | 24% | 71% |
| Completed screening rate with follow-up colposcopy (%) |  | 23% | 22% | 68% |
| Total screening cost^a^ (THB per 100 000 women) |  | 1 145 695 | 696 149 | 1 797 017 |
| Total cancer prevention cost^b^ (THB per 100 000 women) | 49 219 113 | 43 145 345 | 42 034 241 | 37 320 527 |
| Total cancer treatment cost^c^ (THB per 100 000 women) | 6 774 009 | 4 808 614 | 4 768 373 | 3 436 733 |
| Cervical cancer incidence^d^ (cases) | 6214 | 4452 | 4405 | 2841 |
| Death from cervical cancer^d^ (deaths) | 5615 | 4028 | 3985 | 2679 |
| ^a^ Screening cost = screening costs in normal and HPV-infected health states  ^b^ Cancer prevention cost = screening costs + CIN1–3 treatment costs  ^c^ Cancer treatment cost = cervical cancer stage 1–4 treatment costs  ^d^ Calculated based on the total number of Thai women aged ≥ 25 = 24 644 349 women and aged ≥ 30 = 22 253 999 women | | | | |

**Table S5** Model relative outputs

| **Outcomes** | **Clinician-collected samples for  cytology test versus no screening** | **Clinician-collected samples for  HPV DNA testing  versus  clinician-collected samples for  cytology test** | **Self- and clinician-**  **collected samples**  **for HPV DNA testing versus  clinician-collected samples for  HPV DNA testing** | **Unit (per year)** |
| --- | --- | --- | --- | --- |
| **Base-case analysis (women age ≥25 years, screening age: 25-65 years)** | | | |  |
| Incidence case prevented | 7.5 | 0.2 | 7.1 | cases per 100 000 women |
| New case prevented | 1858 | 61 | 1762 | women |
| Cervical cancer death averted | 7 | 0.2 | 6 | deaths per 100 000 women |
| Cervical cancer death averted^a^ | 1676 | 56 | 1483 | deaths |
| Number needed to screen^a^ | 13 264 | 401 082 | 13 990 | women |
| **Scenario analysis (women age ≥30 years, screening age: 30-65 years)** | | | |  |
| Incidence case prevented | 7.9 | 0.2 | 7.0 | cases per 100 000 women |
| New case prevented | 1761 | 48 | 1564 | women |
| Cervical cancer death averted | 7 | 0.2 | 6 | deaths per 100 000 women |
| Cervical cancer death averted^a^ | 1587 | 43 | 1306 | deaths |
| Number needed to screen^a^ | 12 636 | 468 172 | 14 232 | women |
| ^a^ Calculated based on the total number of Thai women aged ≥ 25 = 24 644 349 women and aged ≥ 30 = 22 253 999 women | | | | |

**Supplementary Appendix 5:** Model validation


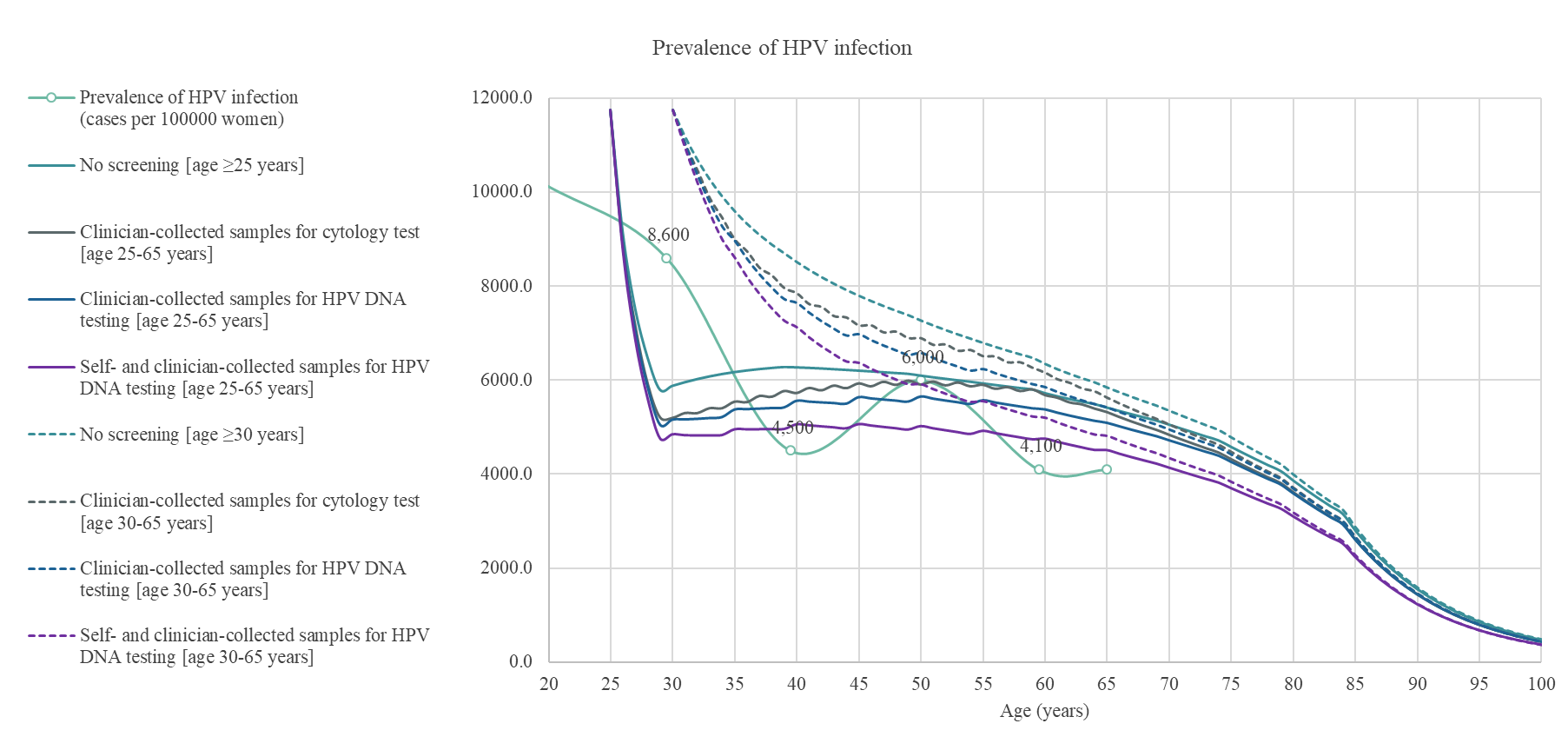


**Figure S2** Prevalence of HPV infection


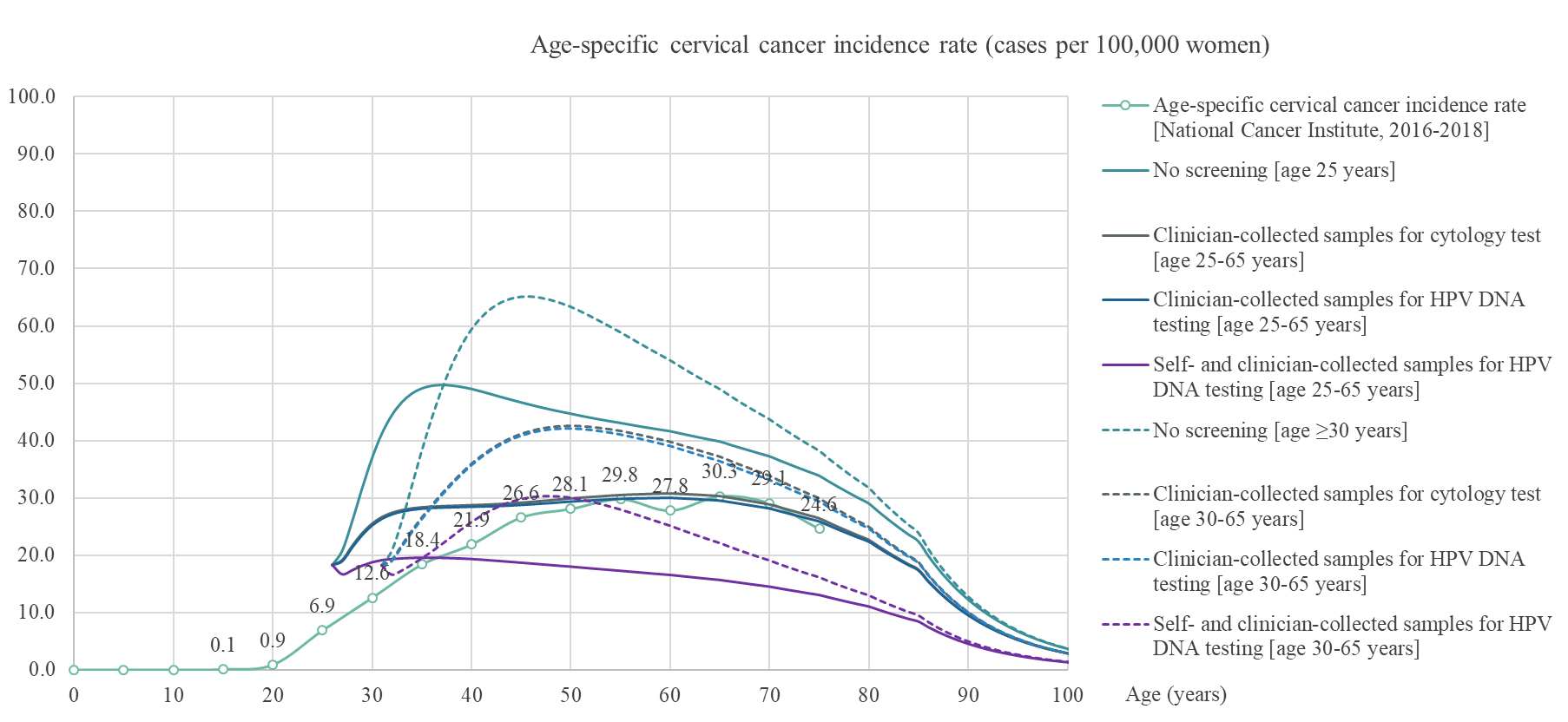


**Figure S3** Age-specific cervical cancer incidence rate

**References**

1. Life tables by country: Thailand. World Health organization. 2019. [Internet]. Available from: https://www.who.int/data/gho/data/indicators/indicator-details/GHO/gho-ghe-life-tables-nmx-age-specific-death-rate-between-ages-x-and-x-plus-n.

2. Termrungruanglert W, Khemapech N, Vasuratna A, Havanond P, Deebukkham P, Kulkarni AS, et al. The epidemiologic and economic impact of a quadrivalent human papillomavirus vaccine in Thailand. PLoS One. 2021;16(2):e0245894.

3. Civil Registration Population Statistics [Internet]. 2022 [cited Jan 1, 2023]. Available from: https://stat.bora.dopa.go.th/stat/statnew/statMONTH/statmonth/#/displayData.
